# Supplementary material for: SpitWorm, a Herbivorous Robot: Mechanical Leaf Wounding with Simultaneous Application of Salivary Components
Source: Plants (Basel). 2019 Aug 31;8(9):318. doi: 10.3390/plants8090318 (PMC6784092; doi:10.3390/plants8090318)
Supplement: Supplementary file 1 [file plants-08-00318-s001.pdf]

Supplementary Materials

SpitWorm, an herbivorous robot: Mechanical leaf wounding with simultaneous application of salivary components

Guanjun Li [1] · Stefan Bartram [1] · Huijuan Guo [2] · Axel Mithöfer [1] · Maritta Kunert [1] · Wilhelm Boland [1]*

[1] Department of Bioorganic Chemistry, Max Planck Institute for Chemical Ecology, Hans-Knöll-Str. 8, D-07745 Jena, Germany; [2] Leibniz Institute for Natural Product Research and Infection Biology – Hans Knöll Institute (HKI), Beutenbergstr. 11a, D-07745 Jena, Germany

* Corresponding author; E-mail: boland@ice.mpg.de

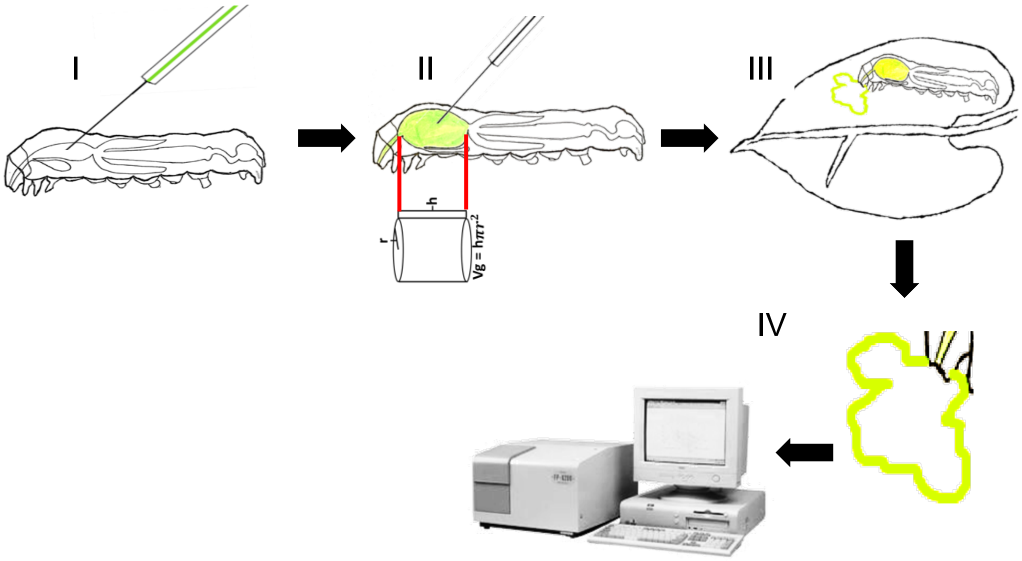

Figure S1. Workflow for determination of OS amount left at the leaf wounding edges. (I) *S. littoralis* larva injected with fluorescent dye into the foregut; (II) *S. littoralis* larva foregut dissected and measured as a cylinder; (III) fluorescent dye solution injected larva fed on *P. lunatus* leaf; (IV) fluorescence dye signal at the wounding area of the leaf being quantified.

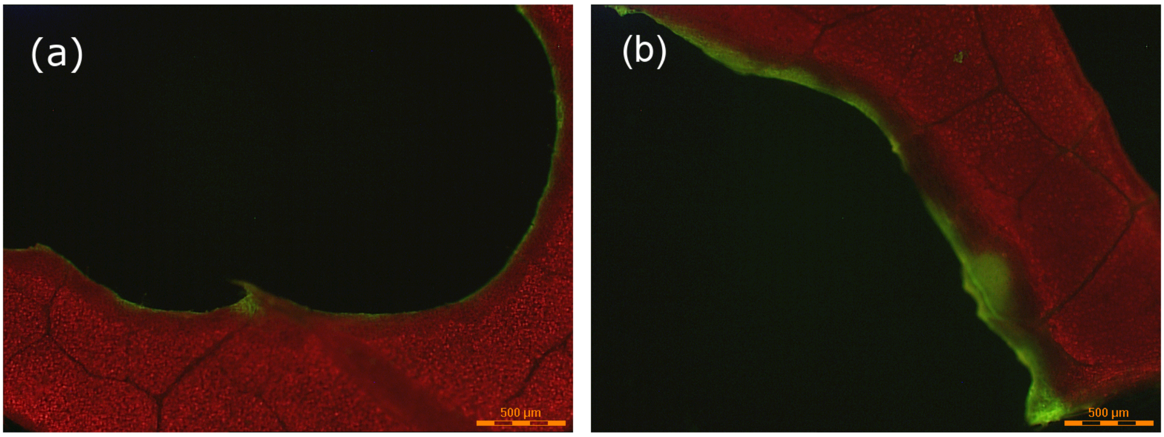

Figure S2. Comparison of fluorescence signals left in plant wounded sites by insects injected with fluorescent dye. (a) Leaf wounded by a *S. littoralis* larva with 1 µL injection; (b) wounded by a larva with 5 µL injection of a solution of Lucifer Yellow in water (1 mg·mL^{-1}).

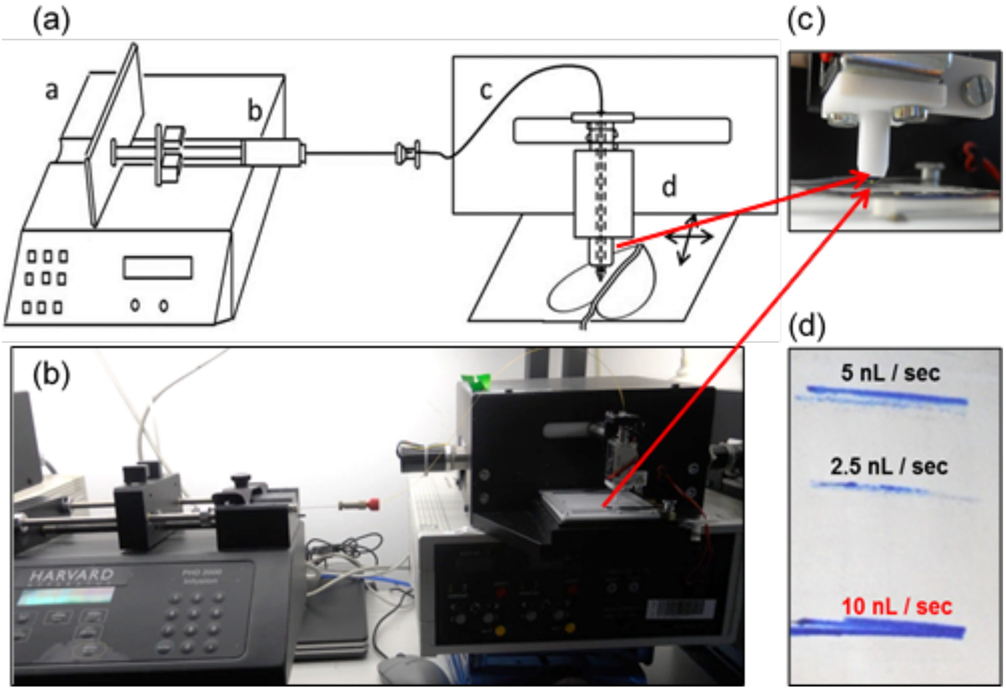

Figure S3. SpitWorm set-up and flow rate optimization. (a) Schematic sketch of SpitWorm: a; Syringe pump to control the delivery rate; b; 100 µL syringe; c; fused silica capillary connecting the syringe to MecWorm through the hollow needle which has a little hole at the tip. d; MecWorm, a system for controlled mimicking the feeding behavior of biting insects. (b) Picture of SpitWorm. (c) An enlarged picture of the 'tooth' of SpitWorm, with an ink droplet at the tip. (d) Ink trails left by SpitWorm at different fluid delivery rates.

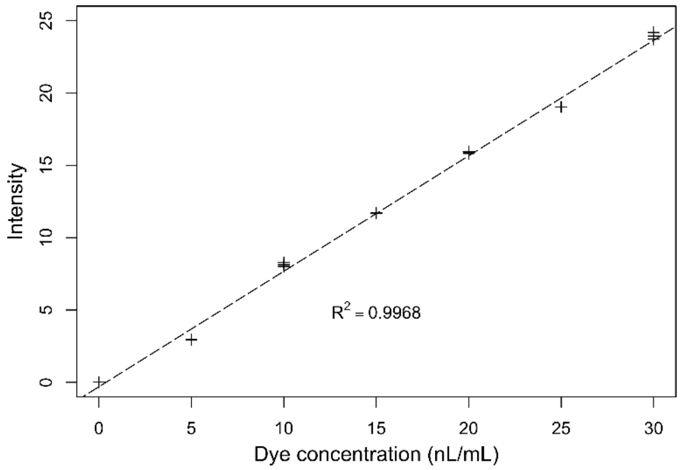

Figure S4. Standard curve. Fluorescent signal intensity of different dilutions (n = 3) of Lucifer Yellow solution (1 mg·mL^{-1}).

Table S1. Analysis of volatile compounds identified and quantified in the headspace after different treatments. Retention indices: RI_{exp}: determined in this study; RI_{lit}: literature data from NIST [36] or § Adams [37]. Multiple comparisons for each compound (n.d., not detected) were performed by one-way ANOVA followed by Tukey's HSD post-hoc test; N, number of replicates; p-values < 0.05 (indicating a statistically significant difference) are set in boldface.

Nr	Compound	Retention index		*Relative amounts*								*p-values*		
				S. littoralis		**MecWorm**		**SpitWorm**		**Control**		***S. littoralis* - SpitWorm**	***S. littoralis* - MecWorm**	**MecWorm - SpitWorm**
		RI_{exp}	RI_{lit}	mean	±SD	mean	±SD	mean	±SD	mean	±SD			
1	α-Pinene	938	937	0.36	0.23	0.23	0.20	0.10	0.15	0.92	0.72	0.0808	0.4924	0.4064
2	Octen-3-ol	980	980	9.55	4.16	33.80	8.94	10.18	5.75	0.46	0.46	0.9849	**0.0000**	**0.0000**
3	3-Octanone	988	986	0.35	0.48	4.64	1.69	1.98	2.87	n.d.	-	0.3380	**0.0040**	0.0931
4	Myrcene	993	991	0.58	0.20	1.15	0.74	0.22	0.26	0.62	0.30	0.4324	0.2146	**0.0167**
5	3-Octanol	996	994	1.74	0.61	3.63	0.83	1.00	1.62	1.30	1.39	0.4995	**0.0342**	**0.0026**
6	Decane	1000	1000	9.62	3.30	0.08	0.17	0.33	0.41	9.93	10.49	**0.0000**	**0.0000**	0.9643
7	Octanal	1004	1003	0.60	0.63	0.95	1.22	0.63	0.96	0.42	0.47	0.9981	0.8466	0.8768
8	(Z)-3-Hexenyl acetate	1007	1005	41.15	17.68	114.52	23.65	25.43	15.92	0.67	0.54	0.3497	**0.0000**	**0.0000**
9	Hexyl acetate	1013	1011	1.30	0.65	7.25	2.63	0.95	0.62	0.05	0.14	0.9317	**0.0000**	**0.0000**
10	(E)-2-Hexenyl acetate	1016	1016	3.27	1.60	20.70	7.26	3.38	2.50	0.02	0.03	0.9991	**0.0000**	**0.0000**
11	o-Cymene	1028	1022	0.02	0.02	0.57	1.00	0.00	0.00	0.00	0.00	0.9979	0.3277	0.2981
12	Limonene	1033	1030	9.90	3.54	0.18	0.15	0.27	0.37	11.27	10.02	**0.0000**	**0.0000**	0.9965
13	(Z)-β-Ocimene	1039	1038	1.22	0.51	5.56	3.40	1.27	1.97	n.d.	-	0.9993	**0.0192**	**0.0208**
14	(E)-β-Ocimene	1050	1049	29.85	11.62	132.88	71.46	37.59	48.59	0.03	0.08	0.9649	**0.0102**	**0.0181**
15	Octanol	1071	1071	1.69	1.39	14.08	7.49	3.90	2.77	0.57	0.80	0.7255	**0.0012**	**0.0072**
16	Linalool	1101	1099	7.24	4.27	15.24	4.72	3.66	2.77	0.66	0.79	0.2894	**0.0061**	**0.0002**
17	Nonanal§	1105	1104	2.98	1.42	5.06	3.11	4.11	2.55	1.85	0.82	0.7149	0.3792	0.8605
18	DMNT	1118	1116	5.25	2.75	52.16	32.35	16.36	23.10	0.00	0.00	0.7276	**0.0186**	0.0937
19	Myroxide	1144	1141	3.52	2.89	5.76	1.84	1.82	0.83	n.d.	-	0.3383	0.2031	**0.0103**
20	(Z)-3-Hexenyl butanoate	1187	1185	3.26	1.23	7.57	2.91	1.86	1.15	0.05	0.14	0.4566	**0.0035**	**0.0002**
21	Methyl salicylate	1202	1192	0.27	0.65	5.99	7.46	0.78	1.01	0.00	0.00	0.9800	0.1412	0.1991
22	Decanal	1207	1206	8.59	5.18	10.41	6.86	14.11	13.98	4.94	1.75	0.5549	0.9514	0.6932
23	2-Phenoxyethanol	1225	1226	1.42	0.65	4.14	2.10	3.16	1.05	1.39	0.88	0.1671	**0.0349**	0.7620
24	(Z)-3-Hexenyl-alpha-methylbutyrate	1234	1234	2.38	1.16	7.43	4.11	3.05	5.04	0.04	0.08	0.9473	0.0635	0.1182
25	(Z)-3-Hexenyl isovalerate	1237	1238	0.87	0.89	6.97	3.38	3.22	2.89	0.00	0.00	0.3756	**0.0111**	0.1889
26	Nonanoic acid§	1264	1267	0.03	0.05	0.88	0.41	0.08	0.14	0.18	0.23	0.9324	**0.0001**	**0.0002**
27	Indole	1301	1295	3.75	1.74	20.30	9.26	10.90	7.64	0.57	0.72	0.2363	**0.0033**	0.1267
28	(E)-3-Hexenyl tiglate§	1326	1315	1.08	0.29	4.56	3.10	2.65	2.64	0.55	0.38	0.5034	**0.0254**	0.2393
29	(E)-2-Hexenyl tiglate	1339	1339	0.06	0.13	0.99	0.90	0.24	0.26	0.07	0.10	0.8509	**0.0118**	**0.0388**
30	α-Ylangene§	1389	1373	0.28	0.24	0.06	0.07	0.33	0.75	0.00	0.01	0.9814	0.6737	0.5540
31	Tetradecane	1400	1400	0.19	0.06	0.45	0.08	0.96	0.38	0.26	0.10	**0.0000**	0.1278	**0.0007**
32	Jasmone	1408	1394	1.10	0.72	2.89	1.65	2.23	3.47	0.06	0.13	0.6422	0.2576	0.7861
33	(E)-β-Caryophyllene	1437	1420	0.23	0.21	1.73	1.29	0.26	0.38	n.d.	-	0.9969	**0.0076**	**0.0090**
34	Geranyl acetone	1457	1453	2.32	1.68	4.35	3.60	5.87	4.01	1.16	0.74	0.1681	0.5979	0.5548
35	β-Ionone	1496	1491	0.02	0.03	1.41	0.30	0.15	0.20	0.00	0.00	0.5899	**0.0000**	**0.0000**
36	Pentadecane	1500	1500	0.82	0.19	0.98	0.63	27.79	13.76	0.80	0.49	**0.0000**	0.9999	**0.0000**
37	δ-Jasmolactone	1503	1518	0.25	0.22	2.61	1.85	2.93	5.66	0.18	0.31	0.3562	0.3338	0.9986
38	TMTT	1583	1577	0.36	0.37	2.69	1.95	1.45	1.31	0.00	0.01	0.4170	**0.0471**	0.4626
	**N**			6		7		6		8	different	4	23	18
											not different	34	15	20
											of total	38	38	38

Table S2. Dimensions of larval foreguts. Lengths (l) and diameters (d) of dissected foreguts were measured. Foregut volume (Vg) was calculated by taking the shape of the foregut as a cylinder.

n	l (mm)	d (mm)	V*g* (mm^3)
1	3	3	21.2
2	5	4	62.8
3	4.3	4	54.0
4	5	4	62.8
5	4	3.8	44.2
mean	4.3	3.8	49.0
sd	0.8	0.4	17.3
